# EIT-Based Fabric Pressure Sensing

**DOI:** 10.1155/2013/405325

**Published:** 2013-02-27

**Authors:** A. Yao, C. L. Yang, J. K. Seo, M. Soleimani

**Affiliations:** ^1^Engineering Tomography Laboratory (ETL), Department of Electronic and Electrical Engineering, University of Bath, Bath BA2 7AY, UK; ^2^Department of Computational Science and Engineering, Yonsei University, Seoul 120-749, Republic of Korea

## Abstract

This paper presents EIT-based fabric sensors that aim to provide a pressure mapping using the current carrying and voltage sensing electrodes attached to the boundary of the fabric patch. Pressure-induced shape change over the sensor area makes a change in the conductivity distribution which can be conveyed to the change of boundary current-voltage data. This boundary data is obtained through electrode measurements in EIT system. The corresponding inverse problem is to reconstruct the pressure and deformation map from the relationship between the applied current and the measured voltage on the fabric boundary. Taking advantage of EIT in providing dynamical images of conductivity changes due to pressure induced shape change, the pressure map can be estimated. In this paper, the EIT-based fabric sensor was presented for circular and rectangular sensor geometry. A stretch sensitive fabric was used in circular sensor with 16 electrodes and a pressure sensitive fabric was used in a rectangular sensor with 32 electrodes. A preliminary human test was carried out with the rectangular sensor for foot pressure mapping showing promising results.

## 1. Introduction

Electrical impedance tomography (EIT) is a fast and cost-effective technique to provide tomographic conductivity image of a subject from boundary current-voltage data. Time difference EIT technique can be used to image conductivity changes in a fabric sensor [1]. When pressure is applied to the fabric patch (the boundary is kept in a frame to maintain a fixed boundary and electrode position), the conductivity of the proposed conductive fabric changes with increasing pressure or deformation of the fabric. Pressure induced shape changes over the sensor area make changes to the conductivity distribution; the change in conductivity distribution leads to the change in current-voltage data in EIT system. EIT system displays the image of the conductivity changes from current-voltage data measured at the boundary of fabric patch. Finally, the pressure distribution could be estimated from the conductivity images.

Pressure mapping can be done with variety measurement methods: capacitive methods [2, 3], piezoelectric method [4–6], inductive method [7–9], and optoelectronic method [10–12]. Pressure mapping techniques have a wide range of applications, including gate and pressure monitoring in biomechanics and touch sensing robotics [13–15]. A change in resistivity of a sensing region can provide an alternative method for pressure mapping and hence EIT has a potential to be used as a pressure mapping imaging tool. Industrial, geophysical, and medical applications of the EIT imaging are well understood. EIT as a pressure mapping imaging method is very new. The original idea of applying EIT-based pressure sensing for pressure ulcers was introduced by Reddy et al. [16] and Fulton and Lipczynski in 1993 [17]. However, experimental trials in [16, 17] were not very successful, partly due to the lack of suitable conductive material for EIT pressure sensor. In [18], a theoretical model was presented for a fabric-based EIT. Electrically active textiles are being developed rapidly in the past few years due to a surge in commercial interest in wearable textiles. Hassan et al. [19] proposed a fabric-based EIT sensor as an artificial robotic skin. Further image quality analyses were carried out on a polymer-based fabric EIT in [20].

In this paper, underlying mathematical framework of EIT-based fabric sensor has been explained and performs various experimental feasibility studies on the use of improved version of EIT-based fabric sensors. The main objective of this study is to show the capability and limitation of the current generation of pressure mapping EIT system and methods. And finally the scientific challenges related to the fabric EIT have been highlighted.

## 2. Mathematical Model

The mathematical framework for the EIT-based pressure mapping imaging can be briefly explained. Let the fabric occupy the two-dimensional domain *Ω* with its boundary ∂*Ω*. On the periphery ∂*Ω* of the fabric, the electrodes are attached *e*_*l*_, *l* = 1,2,…, *L*. A current of 1 mA was injected at a low frequency of 1 kHz with a chosen pair of adjacent electrodes (*e*_*l*_ and *e*_*l*+1_) to generate potential over the fabric *Ω*. Then the resulting potential *U* ^*l*^ satisfies

(1) $$\begin{array}{c} \nabla \cdot \left(\sigma \nabla U^{l}\right)=0\;\text{in}\;\Omega \end{array}$$

with the boundary condition [21]

(2) $$\begin{array}{c} U^{l}+z_{k}\sigma \frac{\partial U^{l}}{\partial n}=V_{k}^{l}\;\mathrm{on}\;\;e_{k},\;\;k=1,2,\ldots ,L,\\ I=\int_{e_{l}}^{}\sigma \frac{\partial U^{l}}{\partial n}dS=-\int_{e_{l+1}}^{}\sigma \frac{\partial U^{l}}{\partial n}dS,\;\;\\ \int_{e_{k}}^{}\sigma \frac{\partial U^{l}}{\partial n}dS=0\;\text{if}\;\;k\neq l,\\ \sigma \frac{\partial U^{l}}{\partial n}=0\;\mathrm{on}\;\;\frac{\partial \Omega }{\cup_{l}^{L}e_{l}}, \end{array}$$

where *σ* is the conductivity distribution of the fabric, *z*_*l*_ is the effective contact impedance at *e*_*l*_, *n* is the unit outward normal vector, and *V*_*k*_^*l*^ is the potential at *e*_*k*_. The distribution of the conductivity is reflected to the measured data:

(3) $$\begin{array}{c} V=\left(V_{1}^{1},V_{2}^{1},\ldots ,V_{L}^{1},V_{1}^{2},\ldots ,V_{L}^{2},\ldots ,\ldots ,V_{L}^{L}\right)\in R^{L\times L}. \end{array}$$

The conductivity perturbation Δ*σ* can be computed by the linearized reconstruction algorithm via

(4) $$\begin{array}{c} \Delta \sigma ={\left(J^{T}J+\lambda L\right)}^{-1}J^{T}\Delta V, \end{array}$$

where Δ**V** is the perturbation of the measured data, *J* is the Jacobian matrix, *L* is the regularization matrix, and *λ* is the regularized parameter. To compute the Jacobian matrix a finite element method (FEM) was used: each element includes a number of cells in knitted structure and *σ* is assumed to be a constant on each element that is the average conductivity of these combined cells that includes air and yarn. The following flowchart summarizes how the pressure is related to the voltage change Δ**V**:

(5) $$\begin{array}{c} \text{Pressure}\Rightarrow \text{Displacement}\Rightarrow \Delta \sigma \Rightarrow \Delta V. \end{array}$$

The inverse problem is to invert this procedure.

## 3. Experimental Results

### 3.1. EIT Hardware and Fabric Sensor

**Figure 1:**
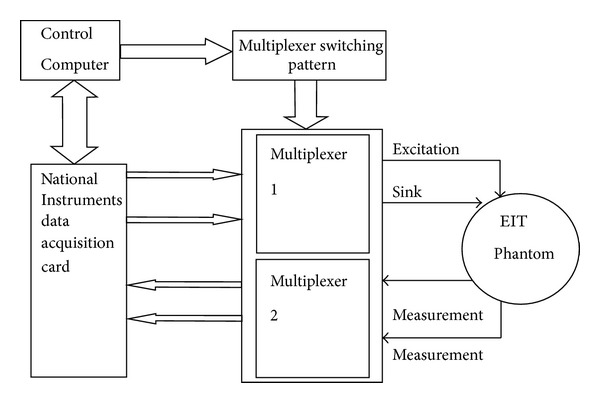
Schematic diagram of the EIT hardware.

A National Instruments LabVIEW Base EIT System was designed for data acquisition and a multiplexer circuit was fabricated for EIT excitation and measurement.  Figure 1 shows the block diagram of the proposed EIT hardware system. The multiplexer 1 supplies excitation current to the electrodes and multiplexer 2 acquires voltage measurements. This is a simple EIT design with an SNR of 40 dB [1].

By acquiring two different sets of voltage measurement data, the reconstruction software is used to do a time difference image reconstruction. To evaluate the repeatability and reliability of the fabric-EIT system, a large number of experiments were carried out.

A number of conductive materials were used in previous studies, for example, silicone-based conductive glue with Ag fillers [18] or polymer-carbon-nanotube composites [20, 22]. In this study an off-the-shelf conductive fabric called EeonTex Conductive Fabric with the model number EeonTex LR-SL-PA-10E5 [23] was used for a circular fabric sensor. This fabric is a knitted nylon/spandex coated with a conductive formulation. The surface resistivity of this fabric is 10^5^ *Ω* per square meter. This is a stretchable sensor showing good elastomeric and electrical properties.

The material used in the square sensor is NW170-SL-PA-1500 [24], which is also developed by the Eeonyx Corporation. It is a microfiber nonwoven material coated with conductive formulation and, by the description on the product information sheet, it is designed for application with a dynamic pressure sensing requirement. The surface resistivity of this material is 1500 *Ω* per square meter ±15%.

### 3.2. Circular Fabric Sensor

The first experiment was designed to analyse the performance of the 16-channel circular fabric sensor. The sensor consists of 3 main parts: a wooden frame, the fabric material, and the electrodes. The frame was constructed by two wooden rings with one on the top of the fabric and the other one at the bottom, and it was used to maintain the fabric patch. Sixteen identical electrodes were placed between two wooden rings and were equally spaced in between. A foam layer was used for protecting the conductive fabric from being damaged.

**Figure 2:**
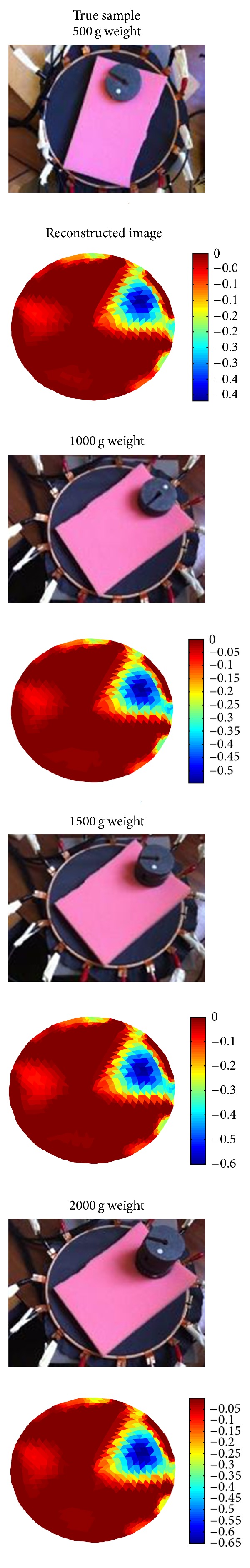
Pressure point at the boundary.

Figure 2 shows the experimental results for a pressure applied close to the boundary of the imaging region. The experiments were done in four steps, starting with 500 g weight with gradually increasing the weight to 2000 g. The reconstruction images show the location where the pressure was applied. The colour-bar scales in the image also show changes relative to the weight.

**Figure 3:**
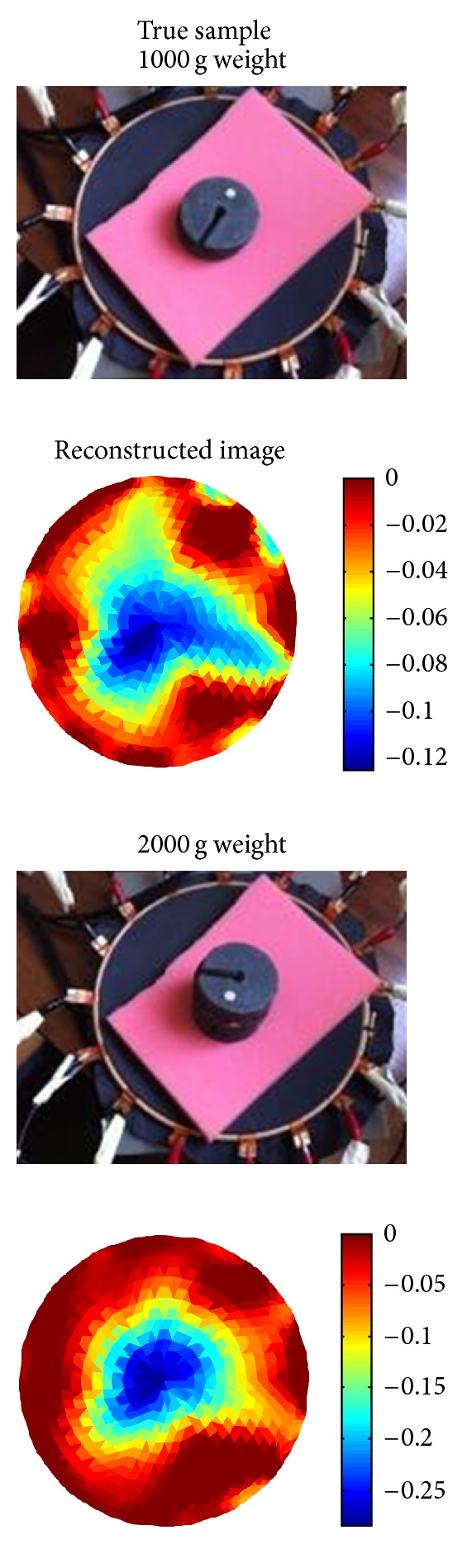
Pressure point at the centre of imaging area.

The second sets of tests were carried out in the middle of the sensor where the sensitivity is at its lowest, shown in Figure 3. Here 500 g sample did not produce a meaningful image. The result for 1000 g weight had shown the pressure location but with distortion due to the fact that the central position is the least sensitive area. An improvement can be observed for larger weight. The scale of colour bar in the images shows changes relative to the weight. Although it is not possible to claim proportionality between image scale and the weight changes, it is promising to see that the scale of reconstructed conductivity follows the weights.

**Figure 4:**
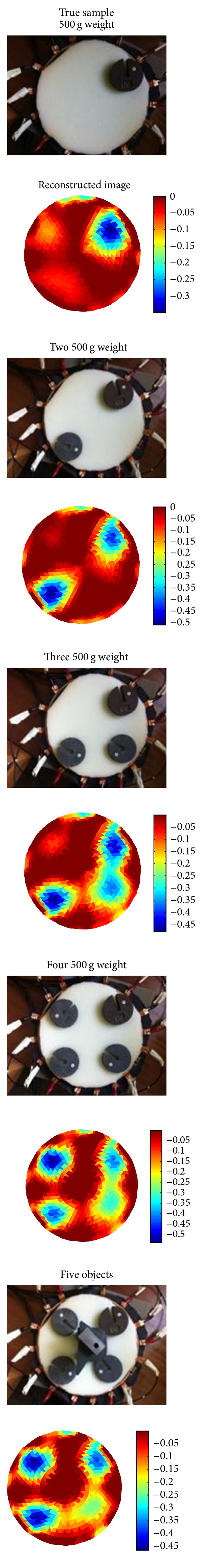
Multiple pressure reconstruction.

It is useful to see how the system performs for multiple pressure locations.  Figure 4 shows reconstruction of 1, 2, 3, 4, and 5 pressure points. It can be seen that the fifth object in the centre in the last experiment could not be reconstructed, which demonstrates lower sensitivity in central imaging area.

### 3.3. Square-Shaped Fabric Sensor

**Figure 5:**
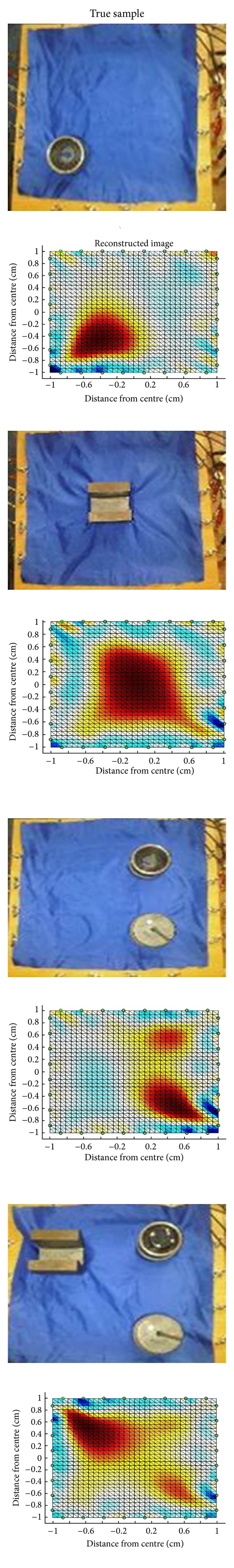
Square sensor.

**Figure 6:**
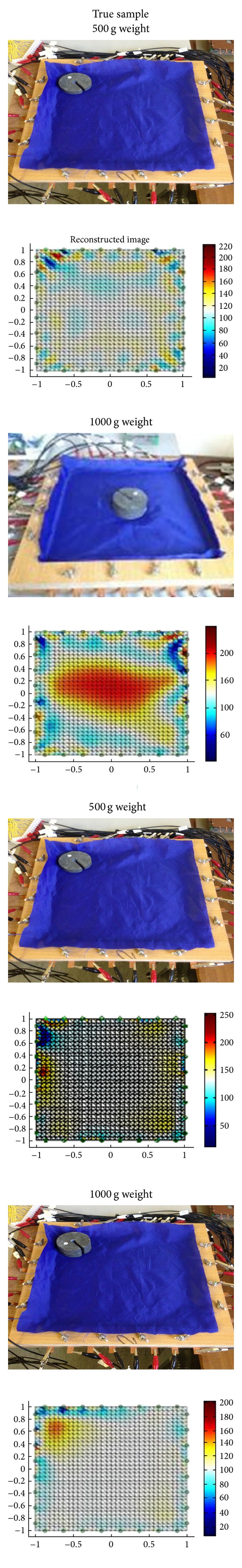
Square sensor with central and boundary pressure points.

A larger area square-shaped sensor was developed for human study. A 32-channel EIT system was used for pressure mapping in a different fabric. W170-SL-PA-1500 was used as a pressure sensitive fabric in this study. The reduced stretch in this sensor reduces potential hysteresis effect since no large scaled deformation could occur when pressure is applied. A wooden frame is used for accurate positioning of the electrodes. The first test was carried to observe the capability of the square fabric sensor of sensing various pressure points. Reconstruction results are shown in Figure 5, similar observations to that of the circular sensor.  Figure 6 shows that 500 g sample could not be detected, while 1000 g sample was detected in central and boundary areas.

### 3.4. Preliminary Human Application Test

**Figure 7:**
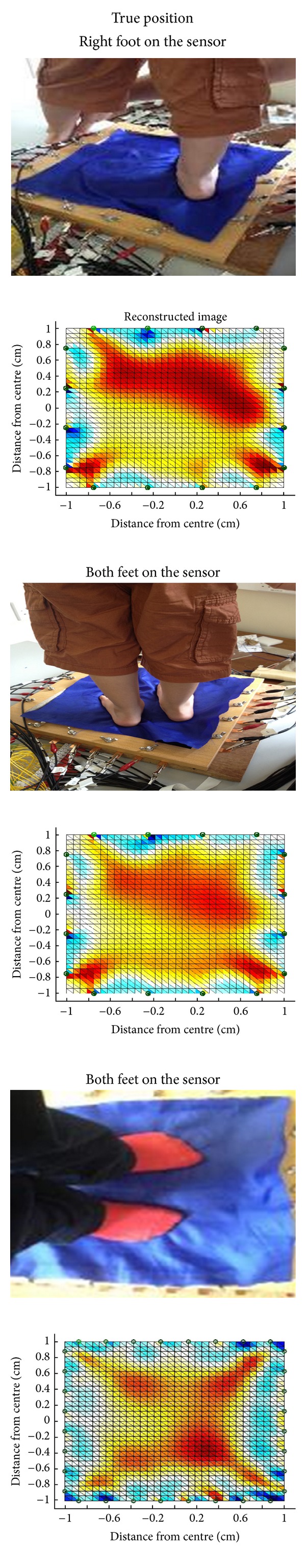
Preliminary human application.

The first human volunteer experiments were carried out as pressure mapping imaging for foot. The sensor does not need to be in direct contact with the person's foot so it is entirely safe to test. The imaging results are shown in Figure 7. The tests were performed on single foot and on both feet of a young volunteer. The first images show the location of right foot of the volunteer. For the second experiment the left foot moved to the sensing area quickly. There is still a strong pressure in the area of right foot and this can be either a hysteresis effect on the sensor or tendency of the volunteer to keep higher level of pressure on their right foot to keep their balance the same way as single foot experiment. The third experiment is when the volunteer moves with both feet to the fabric area. The image shows similar pressure mapping for both feet.

## 4. Discussion and Conclusion

In this paper, EIT-based fabric sensors were presented and tested. For an object in the central areas, the resulting image is less clear when compared to the region closer to the electrodes due to higher sensitivity. In many traditional applications of the EIT, access to central imaging area is not possible. In fabric-based EIT, it is possible to include one or more electrodes inside the imaging region, which could enhance the sensitivity in central imaging area.

The fabric conductivity changes in response to the pressure-induced shape deformation. EIT system provides a real-time imaging of the conductivity distribution change. The conductivity distribution change can be viewed as a nonlinear function of the shape change which can be expressed as a longitudinal displacement. The longitudinal displacement is determined by the pressure distribution, the boundary geometry, and the elastic property of the fabric. The displacement can be computed by solving Poisson's equation with the homogeneous Dirichlet boundary condition. These electromechanical models need to be developed in order to achieve a better understanding of fabric EIT for possible future applications. Circular-shaped fabric sensors have shown promising detectability for single and multiple objects, whereas rectangular-shaped sensors showed some artefacts near corners. The boundary data is relatively sensitive to a perturbation near the electrodes, while it is insensitive to local perturbation away from electrodes. In rectangular-shaped sensors, even if we apply a local pressure away from corners, it may produce an abrupt perturbation near the corners. This problem should be handled via a careful analysis in order to develop robust reconstruction algorithm for visualizing pressure distribution from EIT data. Our future research includes multifrequency EIT-based pressure sensing imaging. Since the modal structure of the fabric-based sensor is not entirely pure resistant, measurement of permittivity can be useful, particularly for capacitive effect in the contact nodes, and the change the deformation of the structure can provide valuable information.
